# Long-term effects of GH therapy in adult patients with Prader-Willi syndrome: a longitudinal study

**DOI:** 10.3389/fendo.2023.1198616

**Published:** 2023-05-26

**Authors:** Graziano Grugni, Alessandro Sartorio, Davide Soranna, Antonella Zambon, Lucia Grugni, Giuseppe Zampino, Antonino Crinò

**Affiliations:** ^1^ Division of Auxology, Istituto Auxologico Italiano, Istituto di Ricovero e Cura a Carattere Scientifico (IRCCS), Piancavallo-Verbania, Italy; ^2^ Experimental Laboratory for Auxo-Endocrinological Research, Istituto Auxologico Italiano, Istituto di Ricovero e Cura a Carattere Scientifico (IRCCS), Milan, Italy; ^3^ Biostatistics Unit, Istituto Auxologico Italiano, Istituto di Ricovero e Cura a Carattere Scientifico (IRCCS), Milan, Italy; ^4^ Department of Statistics and Quantitative Methods, University of Milano-Bicocca, Milan, Italy; ^5^ School of Medicine and Surgery, University of Tor Vergata, Rome, Italy; ^6^ Center for Rare Diseases and Congenital Defects, Fondazione Policlinico Universitario A. Gemelli - Research Institute, Rome, Italy

**Keywords:** Prader-Willi syndrome, adults, GH deficiency, GH therapy, obesity

## Abstract

**Introduction:**

Prader-Willi syndrome (PWS) is a complex disorder resulting from the failure of expression of paternal alleles in the PWS region of chromosome 15. The PWS phenotype resembles that observed in the classic non-PWS GH deficiency (GHD), including short stature, excessive fat mass, and reduced muscle mass. To date, a small number of studies on the long-term effects of GH treatment are available in adult subjects with PWS.

**Methods:**

In this longitudinal study, 12 obese subjects with PWS (GHD/non-GHD 6/6) were treated for a median of 17 years, with a median GH dose of 0.35 mg/day. The median age was 27.1 years. Anthropometric, body composition, hormonal, biochemical, and blood pressure variables were analyzed in all subjects.

**Results:**

Waist circumference was significantly lower at the end of the treatment period (p-value=0.0449), while body mass index (BMI) did not differ significantly. Compared to the baseline, a highly significant reduction of Fat Mass % (FM%) was observed (p-value=0.0005). IGF-I SDS values significantly increased during GH therapy (p-value=0.0005). A slight impairment of glucose homeostasis was observed after GH therapy, with an increase in the median fasting glucose levels, while insulin, HOMA-IR, and HbA1c values remained unchanged. Considering GH secretory status, both subjects with and without GHD showed a significant increase in IGF-I SDS and a reduction of FM% after GH therapy (p-value= 0.0313 for all).

**Discussion:**

Our results indicate that long-term GH treatment has beneficial effects on body composition and body fat distribution in adults with PWS associated with obesity. However, the increase in glucose values during GH therapy should be considered, and continuous surveillance of glucose metabolism is mandatory during long-term GH therapy, especially in subjects with obesity.

## Introduction

Prader-Willi syndrome (PWS) is a rare multisystemic disorder resulting from the lack of expression of paternally inherited imprinted genes in the q11–13 region on chromosome 15. The main genetic mechanisms responsible for PWS are an interstitial deletion of the proximal long arm of the paternal chromosome 15 (del15) (60–70%), maternal uniparental disomy for chromosome 15 (UPD15) (25–35%), or imprinting defects and other chromosome 15 abnormalities (1–4%) (1).

The PWS phenotype is currently thought to be related to a complex hypothalamic dysfunction. The clinical picture is characterized by neonatal hypotonia, poor feeding, and lack of appetite in infancy, followed by hyperphagia leading most subjects to develop morbid obesity from early childhood (if uncontrolled), abnormal body composition, dysmorphic features, behavioral disorders, cognitive impairment and reduced longitudinal growth (2).

Multiple endocrine abnormalities are commonly observed in PWS, including GH/IGF-I axis dysfunction, hypogonadism, central adrenal insufficiency, premature adrenarche, and hypothyroidism (3). The coexistence of an altered GH response to different stimuli and reduced IGF-I levels was found both in children and adult subjects with PWS, with a prevalence of subjects diagnosed with GH deficiency (GHD) that differed between studies in relation to the sample size, weight status, genotypes, and type of GH stimulation test (4). In this light, the PWS phenotype has some common features of classic non-PWS GHD. Apart from short stature, subjects with PWS and GHD are reported to have excessive body fat, decreased muscle mass, reduced muscle strength, hypokinetic cardiac features, impaired bone mineral density, and psychological impairment (5, 6). As a result, GH therapy in children with genetically confirmed PWS without prior demonstration of GHD was approved in the United States in 2000 and in Europe in 2001. Conversely, the Consensus Guidelines for GH therapy in PWS recommended the determination of the presence of GHD after attainment of final height, and in many countries testing before starting the treatment of PWS adults is required (7).

In children with PWS, GH therapy has consistently been shown to improve growth, body composition, metabolic aspects, muscular function, and cognitive development (8). It is notable that these positive effects were observed in children with PWS, both with and without proven GHD (7). As far as adult subjects are concerned, previous data demonstrated a positive effect of GH treatment on body composition, skeletal muscle characteristics, motor performance, heart function, peak respiratory flow, metabolic markers, and psychological well-being, in the absence of major safety issues (6, 7, 9). More recently, a systematic review by Frixou et al. (10) and a meta-analysis by Rosenberg et al. (11) have confirmed that GH administration in adults with PWS was able to significantly improve body composition, without safety concerns. However, most of the studies available were characterized by a short duration, while long-term surveillance of the benefits and risks of GH therapy was mandatory for the PWS population. In this context, data from a cross-sectional study of GH treatment for a median of twenty years showed that all adults with PWS had a normal body composition (12).

With this background, in this longitudinal study, we evaluated the effects of 17 years of GH treatment on body composition, weight status, and metabolic homeostasis in a group of obese adults with PWS. Furthermore, we compared individuals with and without GHD to determine whether GH secretory status predicts metabolic response to GH therapy.

## Materials and methods

### Study population

Twelve obese subjects with a genetically confirmed diagnosis of PWS (seven men and five women; median age 27.1 years, range 18.5–37 years) were consecutively enrolled in the present study. All subjects were Caucasian, showing typical PWS clinical phenotype. Eleven subjects had del15, while UPD15 was found in the remaining individual.

Three men and two women had previously undergone GH treatment, in all cases suspended for at least 2 years before starting the present study. No patient was treated with anti-obesity drugs during the entire study period. Two men had undergone biliopancreatic diversion 10 and 13 years earlier, respectively. All subjects showed normal findings in the main laboratory test, and none had impaired renal or hepatic function or had central adrenal insufficiency, as assessed by the low-dose short synacthen test. During the entire study period, 11 subjects were living with their families at home, while one man was in a mixed residential hostel.

The study was approved by the Ethical Committee of Istituto Auxologico Italiano, IRCCS, Milan, Italy (ref. no. 001C726_2017; acronym: EpidAduPWS), and all subjects and their parents or legal guardians gave their written informed consent to participate in the study. The study was performed in accordance with the Declaration of Helsinki and with the 2005 Additional Protocol to the European Convention of Human Rights and Medicine concerning Biomedical Research.

### Endocrine characterization and study design

At baseline, stimulated GH secretion was evaluated by dynamic testing with a standard GHRH+arginine test. Tests started at 8:30 am after overnight fasting, with the patient recumbent. In addition, basal IGF-1 serum levels were determined. Analyses were performed at the Department of Clinical Chemistry, Istituto Auxologico Italiano of Piancavallo – Verbania. GH levels were measured by chemiluminescence (Immulite 2000, Diagnostic Products Corporation, Los Angeles, CA, USA), as previously described (5). Baseline samples were analyzed for IGF-I determination by using the Liaison XL kit (DiaSorin, Saluggia, Italy), while the more recent samples were analyzed by the chemiluminescent immunometric assay (Immulite 2000, Diagnostic Products Corporation, Los Angeles, CA, USA). IGF-I levels were expressed as SDS, adjusting for age and gender, by using the Apps (IGF1 SD_score), as reported by Chanson et al. (13).

To define the GH deficiency (GHD) status, the BMI-dependent diagnostic cut-off limits of GH peak response (GHp) for subjects with obesity (< 4.2 μg/L) (14), combined with IGF‐I level < − 2 standard deviation score (SDS), were considered.

After baseline examination, the subjects received GH treatment (Genotropin; Pfizer, Rome, Italy) with a median starting dose of 0.30 mg/day for the first month. Subsequently, the dose was adjusted in order to maintain serum total IGF-1 within ±2 SDS from an age-matched reference value to avoid overdosing. At the end of the study, the median GH dose was 0.35 mg/day. All injections of GH were supervised by caregivers. No problems concerning compliance with the injections were reported.

All outcome variables were determined before starting GH therapy and at the end of the study period when the subjects were hospitalized for clinical measurements and functional testing. During the study period, the subjects were regularly followed every 6 months both as in-patients and out-patients, as previously described (15). At discharge from every visit, the subjects and their caregivers received individualized counseling on nutrition and physical activity. The dietary regimen remained unchanged during the follow-up (mean daily energy intake: 1200 Kcal/day). All our subjects performed regular physical exercise (on average 6 hours/week).

### Anthropometric measurements

Physical examination included the determination of height, weight, and waist circumference (WC) by the same trained operators. All subjects were examined wearing light underwear, in fasting conditions after voiding. Standing height was measured using a Harpenden Stadiometer (Holtain Limited, Crymych, Dyfed, UK). Body weight was measured to the nearest 0.1 kg, using standard equipment. WC was determined in a standing position midway between the lowest rib and the top of the iliac crest after gentle expiration, with a non-elastic flexible tape measure.

### Blood pressure measurements and body composition

Diastolic and systolic blood pressure (BP) were measured to the nearest 2 mmHg in the supine position after 5 min rest, using a standard mercury sphygmomanometer with an appropriately sized cuff. The average of three measurements on different days was used.

Dual-energy X-ray absorptiometry (DXA) was used for measurements of fat mass percentage (FM%) and lean body mass (LBM), using a GE-Lunar Prodigy scanner (GE Medical Systems, Milwaukee, WI, USA). No sedation was required.

### Metabolic evaluations

Blood samples were drawn fasting in the morning using venipuncture for determination of glycemia, insulin, hemoglobin A1c (HbA1c)), total cholesterol (TC), high-density lipoprotein cholesterol (HDL-C), and triglycerides (TG). Routine laboratory data were measured using enzymatic methods (Roche Diagnostics, Mannheim, Germany). Insulin resistance (IR) was measured using homeostasis model assessment (HOMA-IR) (16).

### Definitions

Body Mass Index (BMI) was defined as the weight (kg)/height (m)^2^. The BMI cutoff point of 30 kg/m^2^ was used to define obesity (17).

According to the literature (18), metabolic syndrome (MetS) was defined in the presence of three abnormal findings out of the following five parameters: central obesity, high systolic BP and/or diastolic BP, high TG, low HDL-C, and altered glucose metabolism.

### Statistical analysis

Continuous variables are shown as the median, while categorical ones as absolute and relative frequencies. Wilcoxon signed rank sum test was performed to compare the values pre- and post-treatment of the continuous variables as well as the McNemar test for categorical variables. The comparisons between GHD and non-GHD were performed by the Wilcoxon rank test for continuous variables and the Fisher test for categorical ones.

Spearman coefficient (or point-biserial correlation in case of a dichotomous variable) was applied to estimate the correlation between change of anthropometric and body composition variables with selected variables at baseline (age, sex, BMI, weight, GH dosage, IGF-I SDS).

All analyses were performed using the Statistical Analysis System Software (version 9.4; SAS Institute, Cary, NC, USA). Statistical significance was set at the 0.05 level. All p-values were two-sided.

## Results

Six subjects with PWS (2 women) fulfilled the combined criteria for GHD, while the remaining six showed a normal GH-stimulated secretion. IGF-1 SDS values were pathological in 9 subjects.

At baseline, all subjects had central obesity [BMI range 32.8–53.2 kg/m^2^, waist range 87-126 cm (women) and 103-134 cm (men)]. Three women and one man were undergoing sex steroid replacement therapy. Two subjects suffered from central hypothyroidism (1 man) and were biochemically euthyroid on thyroxine substitution. Behavioral abnormalities were present in all subjects, and four of them (3 men) were treated with neuroleptics. MetS was detected in 4 subjects (3 men).

At the end of the study, central obesity was found in all subjects [BMI range 32.4–48.1 kg/m^2^, waist range 93-122 cm (women) and 109-132 cm (men)]. Three women continued sex steroid replacement therapy, while testosterone was withdrawn in the man due to worsening behavior. Five subjects (4 men) were undergoing therapy with neuroleptics. MetS was found in 3 subjects (2 men).

The clinical, laboratory, and instrumental characteristics of our study population, at baseline and after a median of 17 years of GH therapy, are shown in **Tables 1** and **2**, respectively.

**Table 1:** Clinical characteristics of 12 adults with PWS at baseline and after GH therapy.

	Baseline	After GH therapy	p-value
**Age** (years)	27.1	44.65	
**Sex F/M** *n (%)*	5 (42%) – 7 (58%)		
**Genetic** *n (%)* UPD15 del15	1 (8%) 11 (92%)		
**Height** (cm)	155.35		
**Weight** (kg)	97.9	90.7	0.0771‡
**BMI** (kg/m^2^)	42.54	37.01	0.0771‡
**WC** (cm)	124	112	0.0449‡

‡ = Wilcoxon-Mann-Whitney test.

PWS, Prader-Willi syndrome; UPD15, maternal uniparental disomy for chromosome 15; del15, deletion of the paternal chromosome 15; BMI, body mass index; WC, waist circumference.

Data are expressed as median or n (%).

**Table 2:** Laboratory and instrumental characteristics of 12 adults with PWS at baseline and after GH therapy.

	Baseline	After GH therapy	p-value
**IGF SDS**	-3.60	0.45	0.0005‡
**Glycemia** (mmol/L)	4.36	4.97	0.0142‡
**Insulin** (mIU/L)	10.15	9.4	0.6914‡
**HOMA-IR**	2.25	2.34	0.6221‡
**HbA1c** (%)	5.6	5.75	0.7344‡
**Systolic BP** (mm/Hg)	120	120	0.7266‡
**Diastolic BP** (mm/Hg)	80	80	1.0000‡
**TC** (mg/dl)	185	170.5	0.1763‡
**HDL-C** (mg/dl)	48	50.5	0.7744‡
**TG** (mg/dl)	103	107.5	0.8501‡
**FM** (%)	56.45	47.45	0.0005‡
**LBM** (kg)	44.65	48.25	0.0557‡
**MetS** *n (%)*	4 (33%)	3 (25%)	0.3173 ‖

‡ = Wilcoxon-Mann-Whitney test;

‖ = McNemar test.

PWS, Prader-Willi syndrome; SDS, standard deviation score; HbA1c, hemoglobin A1c; BP, blood pressure; TC, total cholesterol; HDL-C, high-density lipoprotein cholesterol; TG, triglycerides; FM, fat mass; LBM, lean body mass; MetS, metabolic syndrome.

Data are expressed as median or n (%).

As expected, IGF-I SDS values significantly increased during GH therapy, thus leading to age-normalized IGF-1 levels in 11 out of 12 subjects. Waist circumference was significantly lower after 17 years of GH treatment (p-value=0.0449), while BMI did not differ significantly.

Compared to the baseline, a highly significant reduction of FM% was observed (p-value= 0.0005). LBM showed a slight increase, but the difference was not significant (p-value= 0.0557).

Both systolic and diastolic BP were unchanged during the study. At baseline, three subjects (2 men) were treated for hypertension, while five subjects (4 men) were receiving anti-hypertensive therapy at the end of the study period.

As far as metabolic parameters are concerned, fasting glycemia was significantly higher at the end of the study (p-value= 0.0142), while insulin, HOMA-IR, and HbA1c values were unchanged. Median TC, HDL-C, and TG values remained stable throughout the study. Considered individually, at baseline one woman had type 2 diabetes mellitus (T2DM) and was treated with insulin, while no other patient showed an alteration of glucose metabolism. During the period of follow-up, the woman with T2DM discontinued insulin therapy. At the end of the study, two women were treated with oral antidiabetic drugs for T2DM, while two additional men had impaired fasting glucose. Normal insulin levels were observed in all subjects at any time during the study. HbA1c values were elevated in the woman with diabetes at baseline and in three additional subjects (2 men) after GH therapy. HOMA-IR was elevated in five subjects (2 women) at the beginning of the study and in four individuals (2 women) at follow-up. At baseline, three subjects (2 women) had slight hypercholesterolemia, five subjects (1 woman) showed low HDL-C levels and hypertriglyceridemia was found in one man. None of them was taking therapy for dyslipidemia. After GH therapy, slight hypercholesterolemia was observed in one woman and low HDL-C values in three men, while one man underwent treatment for hypercholesterolemia.

Finally, no correlation was found between body composition variables (BMI and FM%) and age, sex, BMI, weight, GH dosage, and IGF-I SDS measured at baseline (data not shown).

No major side effects related to GH therapy were found. Transient edema was observed in three subjects within the first month of therapy. In all cases, symptoms resolved with a temporary reduction of GH dose. None of the subjects developed cardiovascular diseases or cancer during the study period.

### Comparison between subjects with PWS (with and without GHD)

The clinical, laboratory, and instrumental data obtained at baseline and at the end of the study in subjects with PWS with different GH secretory statuses are reported in **Tables 3** and **4**, respectively. Both subjects with and without GHD showed a significant increase in IGF-I SDS and a reduction of FM% after GH therapy (p= 0.0313 for all). No other difference occurred at any time during the study.

**Table 3:** Clinical characteristics of subjects with PWS (with and without GHD), at baseline and after GH therapy.

		GHD (N = 6)			non-GHD (N = 6)		GHD vs non-GHD	
	Baseline	After GH therapy	p-value	Baseline	After GH therapy	p-value	p-value at baseline	p-value after GH therapy
**Age** (years)	28.95	48.5		24	37		0.1275‡	-
**Sex Female** *n (%)*	2 (33.3%)			3 (50%)			1.0000†	-
**Genetic** *n (%)* UPD15/del15	1/5			0/6			1.0000†	-
**Height** (cm)	157.15			155.35			0.5752‡	-
**Weight** (kg)	97.9	89.8	0.4375!!!!	99.75	90.7	0.2188!!	1.0000‡	1.0000‡
**BMI** (kg/m^2^)	42.6	35.6	0.4375!!	42.31	39.09	0.2188!!	1.0000‡	0.2980‡
**WC** (cm)	124	112	0.5625!!	125	112	0.0625!!	0.5182‡	0.9361‡

!! = Wilcoxon signed rank sum test; ‡ = Wilcoxon-Mann-Whitney test; † = Fisher test.

PWS, Prader-Willi syndrome; GHD, GH deficiency; UPD15, maternal uniparental disomy for chromosome 15; del15, deletion of the paternal chromosome 15; BMI, body mass index; WC, waist circumference.

Data are expressed as median or n (%).

**Table 4:** Laboratory and instrumental characteristics of subjects with PWS (with and without GHD), at baseline and after GH therapy.

		GHD (N = 6)			non-GHD (N = 6)		GHD vs non-GHD	
	Baseline	After GH therapy	p-value	Baseline	After GH therapy	p-value	p-value at baseline	p-value after GH therapy
**GH peak** (µg/L)	2.8			6.0			0.0051‡	-
**IGF-I SDS**	-4.1	-0.20	0.0313!!	-2.00	1.20	0.0313!!	0.0247‡	0.6304‡
**Glycemia** (mmol/L)	4.3	5.55	0.0625!!	4.5	4.77	0.2500!!	1.0000‡	0.4217‡
**Insulin** (mIU/L)	10.15	7.9	0.4375!!	11.05	11.31	1.0000!!	0.9362‡	0.5752‡
**HOMA-IR**	2.25	1.93	0.5625!!	2.11	2.36	1.0000!!	1.0000‡	1.0000‡
**HbA1c** (%)	5.8	6.1	0.8438!!	5.4	5.6	0.2500!!	0.1697‡	0.4712‡
**Systolic BP** (mm/Hg)	125	120	0.1250!!	120	120	0.6250!!	0.2268‡	0.2406‡
**Diastolic BP** (mm/Hg)	80	80	0.5000!!	70	80	0.5000!!	0.2034‡	0.9233‡
**TC** (mg/dl)	184	175.5	0.2188!!	185	170	0.6875!!	0.9361‡	0.7479‡
**HDL-C** (mg/dl)	40	38.5	1.0000!!	55.5	55	0.6875!!	0.5211‡	0.0916‡
**TG** (mg/dl)	125.5	117	0.6875!!	78.5	103	0.4375!!	0.0776‡	0.5745‡
**FM** (%)	54.75	46.5	0.0313!!	56.45	48.85	0.0313!!	0.9362‡	0.2980‡
**LBM** (kg)	44.5	48.6	0.1875!!	47.55	47.85	0.4375!!	0.6889‡	0.8102‡
**MetS** *n (%)*	3 (50%)	3 (50%)	n.a.‖	1 (17%)	0 (0%)	n.a.‖	0.5455†	0.1818†

!! = Wilcoxon signed rank sum test; ‖ = McNemar test; ‡ = Wilcoxon-Mann-Whitney test; † = Fisher test;

PWS, Prader-Willi syndrome; GHD, GH deficiency; SDS, standard deviation score; HbA1c, hemoglobin A1c; BP, blood pressure; TC, total cholesterol; HDL-C, high-density lipoprotein cholesterol; TG, triglycerides; FM, fat mass; LBM, lean body mass; MetS, metabolic syndrome;.n.a, not applicable.

Data are expressed as median or n (%).

As expected, the comparison between PWS subjects with and without GHD showed that the median GH peak at baseline was significantly higher in individuals with normal GH-stimulated levels (p-value = 0.0051). IGF-I SDS differed significantly between PWS with and without GHD at study entry (-3.5 vs -1.11, p=0.0202) but not after GH therapy (p-value = 0.4712). Comparison of individuals with and without GHD did not reveal any significant difference for the remaining parameters at baseline or after GH treatment.

## Discussion

Adult subjects with PWS associated with obesity displays distinct phenotypic characteristics compared to subjects suffering from non-syndromic obesity. In fact, obesity in PWS is characterized by a marked increase in fat mass associated with a decrease in lean mass, lower trunk-to-appendicular fat mass ratio, and preferential subcutaneous fat distribution (19–22). In addition, these subjects show increased adipocyte volume relative to fat mass and lower expression of genes related to fibrosis and metabolic derangement (22).

GH exerts anabolic effects on several tissues and plays a critical role in regulating metabolism, which results in a marked increase in lipolysis. The benefits of GH therapy in non-PWS adults include improvements in body composition, motor performance, bone characteristics, lipid profile, and quality of life (23). It has been demonstrated that GH treatment yields positive effects on exercise capacity, psychological well-being, and body composition also in adult subjects with PWS, leading to an increase in lean body mass and a reduction in total fat mass (9–11, 24). However, few studies evaluating GH therapy of adult subjects with PWS are currently available, and most of them are of short duration.

The present study longitudinally evaluated the effects of 17 years of GH replacement on body composition in a group of 12 adults with PWS associated with obesity. After a median of 17 years, a significant reduction of FM% and WC were observed. These results were obtained during a multidisciplinary metabolic rehabilitation program, characterized over time by unchanged dietary prescriptions and constant physical activity, under the supervision of a trained staff (15). Our findings confirmed the observations made by other authors, performed with a cross-sectional design, showing a positive effect of long-term GH therapy on the body composition of adults with PWS (12, 25). Differently from the previous studies, however, our investigation enrolled subjects with morbid obesity, demonstrating that the prolonged administration of GH exerted the same benefits in subjects with severe weight excess.

Compared to the general population, there is strong evidence of a reduced life expectancy for subjects with PWS (26). The majority of deaths seem to be the direct or indirect consequences of abnormal body composition. The high fat mass and low muscle mass lead to obesity and its severe complications, including respiratory insufficiency and heart failure, which are associated with the high mortality rate of people with PWS (27, 28). Thus, it is conceivable that some of the comorbidities associated with obesity may be, at least in part, counteracted by the favorable effects on body composition of GH therapy in adults with PWS. However, randomized placebo-controlled studies showing reduced morbidity and mortality in adults with PWS receiving GH therapy are still lacking. For this purpose, a very large number of subjects with PWS is needed.

Our results showed that the improvements resulting from GH administration do not depend on the GH-stimulated secretory pattern. In fact, GH therapy was able to induce a significant reduction of FM% in both subjects with and without GH, as well as an increase in IGF-I levels. Moreover, no significant difference was observed between the two groups for the vast majority of the parameters considered at any time in the study. These findings can probably be explained by the difficulties of confirming GHD in subjects with hypothalamic diseases, such as PWS, as adequate tests are still lacking. In this context, the GHRH-arginine test can lead to falsely normal GH responses, while the insulin tolerance test is often contraindicated because of the cumbersome procedure and potential side effects (11, 29). Altogether, these results support the need for the continuation of GH treatment in adult subjects with PWS, irrespective of their baseline GH status (30).

In our investigation, GH therapy slightly impaired glucose homeostasis, resulting in a fasting glucose increase, while insulin, HOMA-IR, and HbA1c values remained unchanged. Considered individually, glucose metabolism worsened in three additional subjects after GH therapy, whereas the woman with T2DM at baseline discontinued insulin therapy during the study. In this context, close monitoring of glucose metabolism is mandatory during GH treatment in subjects with T2DM or in those predisposed to developing diabetes. However, the worsening of glucose metabolism could be the consequence of the natural clinical history of the disease in subjects with severe obesity, rather than (or in addition) the direct effects of GH therapy. In fact, it has been previously shown that glucose metabolism disorders appear more common in adult subjects with PWS associated with obesity (31). Nevertheless, the lack of a control group is an obstacle in allowing us from addressing the question of whether GH therapy per se causes impaired glucose metabolism. Further longitudinal studies are needed to better understand this crucial point.

The strengths of this study are represented by the long-term duration of GH therapy and its longitudinal design. In addition, all subjects were recruited and followed by a single center, with the same well-trained operators and the same laboratory, which makes the interpretation of the data more reliable than those obtained with a multicenter study. Moreover, body composition was determined using DXA, which represents the gold standard for its evaluation. On the other hand, there are some limitations to our study. The main weakness is related to the small number of the study population, thus resulting in limited strength of the statistical analysis. However, it must be considered that PWS is a rare pathological condition, and enrolment of these subjects is extremely difficult. Another weak point is the lack of an appropriate control group of non-GH-treated patients. In addition, we only considered subjects with PWS associated with obesity, so our data may not be generalizable to all subjects with this syndrome. Furthermore, body fat distribution was evaluated with WC and it can be argued that this might not be the most correct analysis of visceral fat. Nevertheless, WC is commonly used as a surrogate measure of visceral adipose tissue, representing a simple index to monitor changes during interventional studies (32). Finally, our findings were obtained in a tertiary care center with further specialization on PWS, only in Caucasian subjects, and may not be generalizable to other contexts.

In conclusion, our findings show that 17 years of GH therapy has beneficial effects on body composition and body fat distribution in adults with PWS associated with obesity, regardless of whether or not GHD is present. These results seem to support the concept of continuing GH treatment in adults with genetically confirmed PWS without testing for GH secretion, as in pediatric age (30). In our investigation, however, GH therapy slightly impaired glucose homeostasis. The potential clinical consequences of these effects should be considered in the adult setting of PWS, and continued surveillance of glucose metabolism and diabetes risk should be the top priority during long-term GH therapy (7). In this perspective, the relevance of our data remains to be fully established in studies of larger cohorts.

## Data availability statement

The raw data supporting the conclusions of this article will be made available by the authors, without undue reservation.

## Ethics statement

The studies involving human participants were reviewed and approved by Ethical Committee of Istituto Auxologico Italiano,IRCCS, Milan, Italy. The patients and their parents or legal guardians provided their written informed consent to participate in this study.

## Author contributions

GG and AC designed the study. AS dealt with all the administrative aspects and relations with the ethical committee. GG and LG conducted the study. DS and AZ analyzed the data. GG, AS, and AC interpreted the data and drafted the manuscript. AS and GZ contributed to the conceptualization of the findings and provided critical revisions to the manuscript. All authors have read and agreed to the published version of the manuscript. All authors contributed to the article and approved the submitted version.
